# Tumor-Associated Inflammation: The Tumor-Promoting Immunity in the Early Stages of Tumorigenesis

**DOI:** 10.1155/2022/3128933

**Published:** 2022-06-13

**Authors:** Qing Bi, Ji-Yue Wu, Xue-Meng Qiu, Jian-Dong Zhang, Ze-Jia Sun, Wei Wang

**Affiliations:** ^1^Department of Urology, Beijing Chaoyang Hospital, Capital Medical University, Beijing, China; ^2^Institute of Urology, Capital Medical University, Beijing, China; ^3^Third Clinical Medical College, Capital Medical University, Beijing, China

## Abstract

Tumorigenesis is a multistage progressive oncogenic process caused by alterations in the structure and expression level of multiple genes. Normal cells are continuously endowed with new capabilities in this evolution, leading to subsequent tumor formation. Immune cells are the most important components of inflammation, which is closely associated with tumorigenesis. There is a broad consensus in cancer research that inflammation and immune response facilitate tumor progression, infiltration, and metastasis *via* different mechanisms; however, their protumor effects are equally important in tumorigenesis at earlier stages. Previous studies have demonstrated that during the early stages of tumorigenesis, certain immune cells can promote the formation and proliferation of premalignant cells by inducing DNA damage and repair inhibition, releasing trophic/supporting signals, promoting immune escape, and activating inflammasomes, as well as enhance the characteristics of cancer stem cells. In this review, we focus on the potential mechanisms by which immune cells can promote tumor initiation and promotion in the early stages of tumorigenesis; furthermore, we discuss the interaction of the inflammatory environment and protumor immune cells with premalignant cells and cancer stem cells, as well as the possibility of early intervention in tumor formation by targeting these cellular mechanisms.

## 1. Introduction

**Figure 1:**
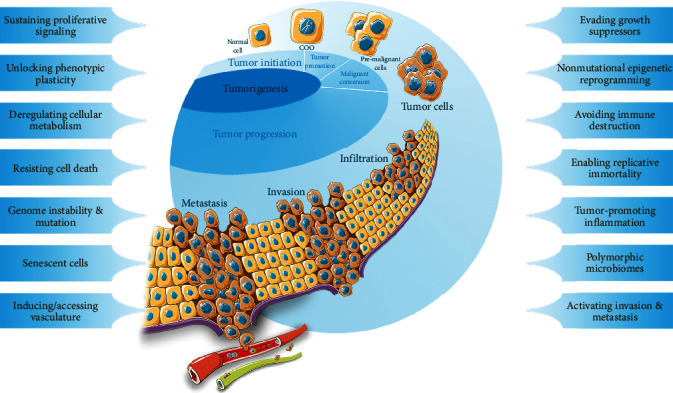
Steps of tumorigenesis and the hallmarks of cancer. A normal cell is transformed into cell-of-origin (COO) after receiving the first oncogenic mutation (tumor initiation). COOs subsequently develop into pre-malignant cells (tumor promotion) and undergo the malignant conversion; they finally enter the tumor progression stage (including tumor infiltration, invasion, and metastasis). During the process, cancers obtain a series of hallmarks and characteristics involving “sustaining proliferative signaling,” “evading growth suppressors,” “resisting cell death,” “enabling replicative immortality,” “inducing or accessing vasculature,” “activating invasion and metastasis,” “deregulating cellular metabolism,” “avoiding immune destruction,” “unlocking phenotypic plasticity,” “nonmutational epigenetic reprogramming,” “polymorphic microbiomes,” and “senescent cells.”

Tumorigenesis, also known as oncogenesis or carcinogenesis, is the transformation of normal cells into cells-of-origin (COOs) [1] after receiving the first oncogenic mutation; it also involves the development of COOs into malignant clones and tumors *via* the selection of the dominant subclones and accumulation of genetic, epigenetic, and transcriptional alterations during subsequent clonal expansion. Tumorigenesis consists of four stages [2–4]: (a) tumor initiation, the initial stage of tumorigenesis, is the stage in which normal cells undergo irreversible genetic alterations under the response of oncogenic factors, thus transforming into COOs with the possibility of malignant transformation; (b) tumor promotion is the period during which COOs clone selectively and transform into premalignant cells under the influence of protumor factors and other specific conditions; (c) malignant conversion is the stage in which premalignant cells start expressing malignant phenotypes; and (d) tumor progression is the final stage of tumorigenesis, in which premalignant cells develop into real tumor cells, obtain a series of new biological characteristics (including sustaining proliferative signaling, evading growth suppressors, resisting cell death, enabling replicative immortality, inducing or accessing vasculature, activating invasion and metastasis, deregulating cellular metabolism, avoiding immune destruction, and unlocking phenotypic plasticity, nonmutational epigenetic reprogramming, polymorphic microbiomes, and senescent cells) [5], and undergo more invasion and metastasis (Figure 1). These characteristics are the result of the superposition of various factors, particularly the tumor microenvironment (TME). The TME is a complex ecosystem composed of tumor cells and other cells in the stroma (endothelial cells, fibroblasts, immune cells, adipocytes, mesenchymal stem cells, *etc*.), as well as the extracellular matrix, blood and lymphatic vessels, and other extracellular components (cytokines, growth factors, hormones, *etc*.) [6–8]. In recent years, the inflammation and immune microenvironment within the TME have been considered as the keys to a breakthrough in the understanding of the TME and the establishment of new cancer therapies [9–11].

Cancer immunosurveillance is an important host-protective mechanism of the immune system that is involved in suppressing tumorigenesis and maintaining cellular homeostasis [12, 13]. However, as early as 1863, Rudolf Virchow recognized the link between tumors and inflammation from the infiltrating lymphocyte in newborn tumors and proposed the hypothesis that “lymphoreticular infiltrate” reflects the origin of tumors from chronic inflammation [14]. As research on cancer-associated inflammation and immunity has advanced, researchers have recognized that excessive inflammation can promote tumor progression as well [15, 16], although localized and limited inflammation is essential to initiate the antitumor immune response. Therefore, cancer immunosurveillance is now more accurately termed cancer immunoediting because tumor immune response can also promote tumor growth and metastasis through, for example, the selection of immunophenotypes [17–19]. Cancer immunoediting consists of three phases: elimination, equilibrium, and escape [20, 21]. While immunoediting primarily plays an “elimination” role in the early stages of tumorigenesis, we nevertheless believe that there is a fraction of immune cells that can help tumor cells enter the “equilibrium” or even “escape” phase and initiate tumor formation.

Published reviews have extensively described how immunity promotes progression and metastasis in the late stages of tumorigenesis [18, 19]; therefore, we aim to understand the mechanism by which these protumor immune cells help premalignant transformed cells survive the “elimination” phase in the earlier stages. Accordingly, in this manuscript, we review the existing evidence regarding the involvement of immune cells in the early stages of tumorigenesis, discuss the potential mechanisms, and present our thoughts on interventional therapies.

## 2. Immune-Driven DNA Damage and Repair Inhibition Promote Tumor Initiation

DNA damage and mutations are important bridges between chronic inflammation and tumor initiation [22–24]. One example of this is the recruitment and expansion of inflammatory cells in the prostate of patients with chronic prostatitis; this can promote DNA double-strand breaks in prostate epithelial cells as well as the activation of androgen receptors, which is one of the important inducements of prostate cancer [25]. During inflammation, epithelial and immune cells, especially neutrophils and macrophages, fight pathogens and stimulate tissue repair and regeneration by producing reactive oxygen and nitrogen species (RONS) [26]. The excessive increase of these chemicals will result in the saturation of the antioxidant system, and the oxidative stress will lead to a variety of biological macromolecules, including nucleic acids, proteins, and lipids, being damaged; however, DNA is the most sensitive target [27]. RONS can induce oxidation, deamination, halogenation, lipid peroxidation-derived adducts, and single- or double-strand breaks, all of which lead to DNA damage and mutations [26]. In addition to being secreted extracellular, reactive oxygen species (ROS) are also produced intracellularly due to inflammatory factors, such as TNF-*α*, IL-1*α*+*β*, and IFN-*γ* [28, 29].

Following the damage and mutation, DNA repair is essential for survival and can be effective in preventing tumorigenesis [30–32]. Nevertheless, the persistence of RONS can adversely affect DNA repair. For instance, S-nitrosoglutathione generated by the reaction of nitric oxide with glutathione can engage in S-nitrosylation [33], resulting in an imbalance of base excision repair which is one of the most crucial DNA repair mechanisms [34]. Furthermore, S-nitrosoglutathione reductase (GSNOR) is considered to be a key protein in maintaining the homeostasis of the S-nitrosylation, and GSNOR-deficient mice exhibit massive S-nitrosylation and proteasomal degradation of the key DNA repair protein *O*^6^-alkylguanine-DNA alkyltransferase, thereby inhibiting the DNA repair system; however, this effect can be blocked by the inhibition of inducible nitric oxide synthase (iNOS) [35, 36]. In addition, the S-nitrosylation reaction has been shown to lead to a decrease in the activity of ligase, which is responsible for the most critical step in DNA repair [37].

DNA damage [38–41] or the endogenous accumulation of genomic instability [42] can also induce or exacerbate inflammation, which is an immune response aimed at clearing the damaged cells. However, prolonged unresolved inflammation will inevitably lead to a vicious cycle of disease progression *via* the mechanisms described above.

## 3. Immune Trophic/Supporting Signals Drive the Early Proliferation and Dissemination of Premalignant Cells

From the early stages of tumorigenesis, premalignant transformed cells can already induce an inflammatory response through the recruitment of innate immune cells, thus promoting their appreciation and triggering the subsequent metastatic spread of cancer. Feng et al., who implanted transformed cells into zebrafish, reported that these premalignant cells as well as paracancerous epithelial cells regulated leukocyte activation and recruitment *via* dual oxidase-mediated H_2_O_2_ signaling to promote the growth and progression of premalignant cells; blocking leukocyte maturation (by knocking down *pu.1* and *gcsfr1*) could limit this process [43]. Subsequent studies on zebrafish confirmed that this process is involved in a variety of tumorigeneses, and new studies have shown that there are more signal pathways involved. In skin and brain tumor models, neutrophil recruitment was dependent on CXCL8 (IL-8)/CXCR1+2 signaling [44, 45], while macrophages/microglia were recruited *via* CSF-1 (M-CSF) and CXCL12b (SDF1b)/CXCR4b pathways [46]. These recruited leukocytes could release COX2 and microsomal prostaglandin E synthase (mPGES) signaling to mediate PGE_2_ production and promote the growth of premalignant cells through EP1 receptors [47]. Confocal in vivo imaging also confirmed that microglia in the zebrafish brain were in prolonged contact with premalignant cells and underwent highly dynamic changes (constant expansion and contraction); it also showed that blocking Ca^2+^/ATP/purinergic receptor P2Y12 (P2RY12) signaling (by interfering with Ca^2+^ levels, inhibiting ATP release, or knocking down P2RY12) reduced the interaction of premalignant cells with microglia and impaired the proliferation of the former [48]. Moreover, TGF-*β* [49], TNF-*α*, and caspase-a (the zebrafish homolog of human caspase-1) [50] have also been shown to play a role in recruiting leukocytes, promoting inflammation production, and supporting premalignant cells in the zebrafish liver tumor model.

Although similar effects of premalignant cells have not been much reported in mammalian models, some indirect evidence for a supportive role of immune cells still exists. Premalignant cells can recruit and activate CD206/Tie2 macrophages *via* CCL2 in MMTV-HER2 breast cancer mice; these activated macrophages disrupted the intercellular E-cadherin junctions by producing Wnt-1, providing the conditions for the dissemination of early breast tumor cells [51]. Mouse breasts exhibited preneoplastic changes (including increased ductal branching, hyperplasia, and dysplasia) when overexpressing CSF-1 and/or its receptors [52]. Carper et al. observed that immune cell infiltration resulted in the development of premalignant lesions in the HPV16(+) head and neck squamous cell carcinoma mouse models they generated [53]. Similarly, *K-ras^G12D^* mutant mice developed an accumulation of inflammatory cells (especially neutrophils and macrophages) in their lungs, which promoted the formation of lung cancer by inducing the chronic obstructive pulmonary disease-like airway inflammation; limiting the recruitment of neutrophils (by inhibiting CXCR2) or neutrophil-depletion significantly reversed the formed tumors, hindered tumor progression, and forced most tumors to remain at early stages [54, 55]. The massive infiltration of MDSCs and macrophages in the intestinal epithelium also accelerated the tumorigenic process of inflammation-driven tumors due to the overexpression of CXCR4 [56]. These macrophages may play a partial trophic role through the secretion of IL-6 [57]. Monocyte chemotactic protein 1 (MCP-1) is considered to be the most important chemokine that recruits macrophages to the TME; the deletion of MCP-1 leads to a reduction in the number and size of colorectal polyps in ApcMin/+ mice [58], similar to the consumption of macrophages [59].

Innate immune cells from other inflammation sites can also be involved in the support of premalignant cells. In the zebrafish model, periwound neutrophils rapidly moved around premalignant cells, which played a nutritional and proliferative role through interaction and PGE_2_ [60]. Notably, Hayes et al. proved the protumor effect of basophils by inducing oncogenic mutations in mouse epithelial cells (though not enough to promote tumor growth) and TPA- (a protein kinase C activator-) induced inflammation, which was not reported in zebrafish models. Skin inflammation upregulated the expression of CXCR4 on the surface of IgE-bearing basophils dependent on thymic stromal lymphopoietin and IL-3; these basophils were subsequently recruited by CXCL12 (binding to CXCR4) and activated by Fc*ε*RI-signaling to promote the growth and tumorigenesis of epithelial cells containing oncogenic mutations partially through histamine [61].

## 4. Immunosuppressive Cell-Induced Immune Escape in Early Tumorigenesis

The recruitment of immunosuppressive cells by tumors occurs throughout tumorigenesis, from the emergence of DNA damage and the formation of transformed cells to the development of infiltration and metastasis. These immunosuppressive cells not only promote tumor proliferation by secreting cytokines but also help tumors evade immunosurveillance by disrupting antigen presentation, inhibiting the proliferation and activation of T and B cells, and/or suppressing the cytotoxicity of cytotoxic T lymphocytes (CTLs) and natural killer (NK) cells; thus, they create a “sanctuary” called suppressive tumor immune microenvironment (TiME) where tumor cells can avoid their “enemies.” Indeed, the formation of suppressive TiME may precede the process of tumorigenesis [62], and the immune escape occurred before tumor invasion [63]. The immunosuppression and tolerance of the TiME mainly derive from tumor-associated macrophages (TAMs), regulatory T cells (Tregs), and myeloid-derived suppressor cells (MDSCs) [64, 65]. Macrophages may be the immune cells that initially infiltrate into the TME and subsequently recruit other immune cells such as neutrophils and monocytes by secreting chemokines [62, 66]. There are two main polarization states of macrophages: the M1 type (involved in the inflammatory response, pathogen clearance, and antitumor immunity) and the M2 type (with protumor properties). TAMs are more similar to M2 polarized macrophages, which can promote tumor immune escape by suppressing dendritic cells (DCs) and CD8^+^ T cells; these effects are mediated by IL-10 or the expression of the negative costimulatory molecules like programmed death-ligand 1 (PD-L1) [67]. Tregs, rather than CD8^+^ T cells, are the main responsive T cells in the early stages of tumor formation and are also involved in tumor immune escape [68]. Similar to TAMs, Tregs suppress the activation and proliferation of antigen-specific effector T cells (Teffs) *via* secreting IL-10 and TGF-*β*, or by expressing programmed death 1 (PD-1)/PD-L1 and cytotoxic T lymphocyte antigen 4 (CTLA-4) [69]. Furthermore, Tregs can undergo a TAM-induced recruitment by CCL22 [67]. MDSCs can impair the function of DCs, NK cells, and CTLs by overexpressing RONS, releasing IL-6 and IL-10, or depleting L-arginine (L-Arg), thus facilitating tumor escape [70]. Moreover, tumor-associated neutrophils (TANs) secrete arginase 1 (Arg-1) to degrade extracellular L-Arg, involved in the functional suppression of CD8^+^ T cells [71]. In this section, we mainly focus on published evidence related to immune escape during the early stages of tumorigenesis.

### 4.1. Macrophages

Direct evidence suggesting the involvement of macrophages in immunosuppression during the early stages of tumorigenesis is limited. Medler et al. reported that urokinase-expressing macrophages regulated C3-independent C5a release during tumor promotion in K14-HPV16 transgenic mice; this, in turn, regulated the protumorigenic properties of macrophages (high in C5aR1 expression), including the inhibition of the cytotoxic activity of CD8^+^ T cells [72]. Another study observed that M2 macrophage-infiltration was higher in cervical precancerous lesions compared to normal tissues [63], which is consistent with the results of a study conducted on a similar stage of squamous cell lung carcinoma; these lesion-associated macrophages, similar to TAMs, have an immunosuppressive effect [73].

### 4.2. Tregs

There is some evidence that Tregs are also involved in the immune escape during the early tumorigenesis stage. For instance, the induction of oncogenic *Braf^V600E^* and loss of *Pten* in melanocytes promoted the expression of CCR4, which induced the inhibition of CD8^+^ T cell-mediated immunosurveillance autochthonous melanoma tumorigenesis in mice by recruiting Foxp3^+^ Tregs [68]. In another study on the administration of azoxymethane (AOM)/dextran sulfate sodium salt- (DSS-) induced colon cancer in mice, diphtheria toxin (DT) injection (for the depletion of Foxp3^+^ Tregs) after the last DSS cycle increased the number of CD62L^low^ CD8^+^ Teffs, accompanied by enhanced cytotoxic activity (upregulated-expression of IFN-*γ* and granzyme B), in the colon of mice, as well as a significant reduction in the distribution of tumors in the colon, which could be completely attenuated by CD8^+^ T-cell-depleting antibodies [74]. This process is likely regulated positively by IL-33 and IL-17 and negatively by IL-10. IL-33/suppression of tumorigenicity 2 (ST2) signaling activates CD4^+^ Foxp3^+^ Tregs, promotes their accumulation in the colon, and accelerates AOM/DSS-induced colonic carcinogenesis, which may occur because blocking the IL-33/ST2 pathway reduces the IL-17 production by Foxp3^+^ Treg cells, thereby altering the inflammatory signaling in the TME and inhibiting Th17 differentiation. Knocking down of the ST2 receptor on Tregs in mice engendered a reduction in Tregs infiltration, accompanied by the accumulation of CD8^+^ T cells; this resulted in fewer and smaller induced tumors, with significantly delayed progression [75]. Conversely, a study on *Il10^fl/fl^*/*FIC* mice have shown that the deletion of IL-10 from Tregs triggered more severe inflammation, leading to enhanced tumor formation and growth [74]. It has also been reported that the depletion of Tregs can also protect mice from methylcholanthrene- (MCA-) induced fibrosarcoma in an NK cell-dependent manner and that the complete depletion of Treg cells can even cure some tumor-bearing mice [76]. While this evidence suggests that Tregs play an important role in promoting tumorigenesis in model mice by suppressing the effector functions of CD8^+^ T cells and NK cells, certain controversies remain. For example, Martinez et al. found that the ablation of Tregs in mice with carcinoma *in situ* increased the number and size of breast tumors, accelerating their transformation into invasive cancer [77]. Furthermore, the injection of DT during the DSS cycles exacerbated inflammation, leading to more deaths [74]. Similarly, the deletion of Tregs reduced tumorigenesis but enhanced colitis in *Bacteroides fragilis*-colonized *C57BL*/*6 Foxp3^DTR^* mice due to the mucosal cytokines shifting from IL-17 to IFN-*γ* [78].

### 4.3. MAIT Cells

Mucosal-associated invariant T (MAIT) cells, a type of unconventional T cells that rely on MHC class I-related protein 1 (MR1) for their development and function, have always been known for their antimicrobial properties [79]; however, recent reports have revealed their negative role in tumor immunity. MAIT cells exist widely in various TMEs and can be activated by the MR1 of tumor cells *via* IL-17, thereby inhibiting the effector functions of NK cells and/or CD8^+^ T cells (including IFN-*γ* release and degranulation) to promote tumor initiation, proliferation, and metastasis [80, 81]. In an experiment involving fibrosarcoma induced by MCA, long-term monitoring results showed that *mr1*^−/−^ mice exhibited stronger resistance to MCA than wild-type mice, demonstrating that the lack of MAIT cells can provide better protection against tumor formation [80].

### 4.4. MDSCs

Early in tumorigenesis, the local C5a can recruit MDSCs by binding to C5aR1, thereby impairing the proliferation and function of CD8^+^ T cells, creating a pretumor immunosuppressive microenvironment and ultimately promoting AOM/DSS-induced mice colorectal carcinogenesis [82]; this has also been found in melanoma mice [83]. In another study, the deletion of *Ripk3* in MDCSs promoted the activation of the NF-*κ*B/COX-2/PGE_2_ axis, induced the infiltration of granulocytic MDSCs (G-MDSCs), and facilitated colorectal carcinogenesis, whereas the targeted inhibition of COX-2 and EP2 attenuated the immunosuppressive activity and oncogenic effects of MDSCs [84]. However, Jayakumar and Bothwell reported that *Ripk3* deficiency in intermediate MDSCs (I-MDSCs) was protective against inflammation-induced colorectum cancer, which seems unlikely [85]. Furthermore, Zhou et al. found, in the induction of mice lung tumorigenesis, that MDSCs and macrophages could both directly contact and kill CD4^+^ and CD8^+^ T cells by expressing Fas ligand, perforin, and granzyme A; they could also indirectly suppress CD4^+^ Th 1 cells and CD8^+^ T cells by promoting the development of Tregs and suppressing DCs *via* secreting IL-10, TGF-*β*, and NADPH oxidase 2 (NOX2) [86]. MDSCs are equally important in the progression of precancerous lesions. The restriction of MDSCs in the ApcMin/+ adenomatous polyposis mouse model effectively enhanced the cytotoxic function of CD8^+^ T cells and inhibited the progression of polyps [87]. Elevated levels of MDSCs, which perform immunosuppressive functions, were also found in the peripheral blood of a few human patients with precancerous lesions, including colon adenoma and intraductal papillary mucinous neoplasm [88].

### 4.5. B Cells

B cells are key to humoral immunity and play an important role in limiting infection and tumor development. However, as research has progressed, their role in tumorigenesis has become controversial. In particular, recent evidence suggests that B cells may even limit the antitumor function of CD8^+^ T cells by secreting the neurotransmitter gamma-aminobutyric acid to promote the differentiation of monocytes into IL-10^+^ macrophages [89]. In mice with inflammation-induced colon cancer, the depletion of neutrophils engendered B-cell infiltration, and the inhibition of B cells significantly reduced the tumor load, size, and aggressiveness [90]. Similarly, B cells acted synergistically with Fcr*γ*^+^ myeloid cells to promote pancreatic ductal adenocarcinoma tumorigenesis [91]. It has been reported that tumor cells induce the production of a specific B cell population called CD25^+^ B220^+^ regulatory B cells (Bregs); these Bregs induced the conversion of CD4^+^ T cells into Foxp3^+^ Tregs by secreting TGF-*β*, thus exerting an immunosuppressive effect and promoting the development of metastasis [92]. The same effect was also confirmed in CD19^+^ IL-10^+^ Bregs obtained from human tongue squamous cell carcinoma tissue [93].

## 5. Immune-Enhanced Characteristics of Cancer Stem Cells (CSCs)

CSCs, sometimes understood as tumor-initiating cells (TICs), are a class of cancer cells with self-renewal, pluripotency, and high oncogenicity, which have been demonstrated to be a key tumor-initiating subpopulation in several cancer types [94]. The results of single-cell sequencing showed that some colonic precancer subtypes already exhibited high stemness during the premalignant stage [95]. Noticeably, inflammatory signals could induce the dedifferentiation of epithelial cells into CSC-like cells, leading to colorectal carcinogenesis [96]. Stem cells are thought to survive in microenvironments called niches, which are composed of fibroblasts, immune cells, endothelial cells, extracellular matrix components, cytokines, growth factors, and suchlike [97, 98]. The niches can not only protect stem cells from depletion but also limit their overproliferation. By providing intercellular contacts and secreted factors, niches can determine the differentiation direction of stem cells and regulate their participation in tissue generation, maintenance, and repair [98, 99]. Several reports suggest that the immune cells previously present around CSCs can activate the transformation of CSCs into a more active and malignant state. For example, *in vivo* injection of TAMs promoted the expression of the hepatocellular carcinoma (HCC) stem cell annotator CD44 in mice, which was consistent with the results of the coculture of TAMs and CSCs *in vitro* (the proliferation of CSCs was promoted) [100]. Lu et al. depleted endogenous macrophages while implanting CSCs in mice, resulting in the near-complete prevention of tumorigenesis [101]. In another study, TANs secreted bone morphogenetic protein 2 and TGF-*β*2, thus facilitating the dedifferentiation of HCC cells into CSCs; these TANs can also stimulate CSCs to recruit more TANs by upregulating NF-*κ*B signaling and CXCL5 secretion leading to the formation of a vicious cycle [102]. Furthermore, CSCs have been shown to shape the niches that meet the needs of their progression by inducing immune cell recruitment and conversion to pro-CSC subtypes. Colorectal CSCs (CRCSCs) were found to secrete CXCL1+2 to attract CRCSC-primed neutrophils, thereby promoting tumorigenesis in CRC cells *via* IL-1*β*; eliminating these neutrophils reduced the carcinogenicity of CRCSCs [103]. Similarly, the cholangiocarcinoma stem-like subset recruited circulating monocytes into the niches and induced their differentiation into CSC-associated TAMs by releasing factors such as IL-13, IL-34, and osteoactivin [104]. These “educated” tumor-infiltrating macrophages have also enhanced the initiation properties of CSCs [105]. CSC-released CCL5 induced the infiltration of Tregs, such as breast cancer [106] and ovarian cancer [107]. These Tregs were confirmed to enhance the stemness and tumorigenicity of breast cancer cells and CSC-like populations [106]. Indeed, those recruitment signals mentioned in the previous sections, including CXCL8/CXCR1+2 and CXCL2/CXCR2 signaling pathways, are also important for CSCs [108–110]. Blocking of these signals predictably resulted in significant inhibition of the characteristics of CSCs [109, 111, 112]. G-CSF and CXCL5 overexpression results in more CSCs in cervical cancer [113] and prostate cancer [114], respectively, by attracting MDSCs.

Although we have known these key recruitment signals, the mechanisms by which those pro-CSC immune cells enhance the characteristics of CSCs still have a limited understanding. In a study on HCC, TAM-released IL-6 promoted the progression of hepatocellular carcinoma stem cells through STAT3 signaling [100]. Notably, the same signaling pathway has also been demonstrated in a study of MDSCs and breast cancer [115], as well as Tregs and glioma [116]. Another study found that a type of CD4^+^ T cells can also use the IL-22-mediated activation of the STAT3 transcription factor to promote colorectal cancer stemness *via* inducing the H3K79 methyltransferase disruptor of telomeric silencing 1-like [117]. Furthermore, hypoxia upregulated IL-17 expression in Foxp3^+^ Tregs and subsequently drove the conversion of bone marrow-derived mononuclear cells into TICs through Akt and MAPK activities [118].

## 6. The Activation of Inflammasomes in Tumorigenesis

Inflammasome, an oligomeric protein complex proposed by the Jürg Tschopp research group in 2002 [119], consists of receptors (nucleotide-binding domain-like receptors [NLR] or absent in melanoma 2-like receptors [ALR] or Pyrin) and an enzyme component (caspase-1). Furthermore, there is a junction molecule called ASC (apoptosis-associated speck-like protein containing a caspase recruitment domain) in most inflammasomes [120]. As a key regulator of innate immunity, inflammasomes are primarily assembled in immune cells, particularly in macrophages and dendritic cells, and also expressed and activated in nonhematopoietic cells, such as epithelial cells [121]. Inflammasomes are activated by recognizing the pathogen-associated molecular patterns (PAMPs) or damage-associated molecular patterns (DAMPs) released by infected cells, damaged tissues, and tumors; this results in the caspase-1-dependent secretion of inflammatory cytokines IL-1*β* and IL-18, thereby inducing pyroptosis [120–122].

The available reports demonstrate that the activation of inflammasomes was involved in the early links of tumorigenesis (Table 1). For example, in *gp130*^F/F^ spontaneous intestinal-type gastric cancer mice models, the genetic ablation of *Asc* resulted in reduced caspase-1 and NF-*κ*B activity, decreased expression of mature IL-18, and increased caspase-8–like apoptosis in the gastric epithelium; consequently, tumorigenesis was suppressed, which was consistent with the ablation of the *Il18* gene [123] and *Aim2* deficiency [124]. In *H. pylori*-infected mice, overexpressing IL-1*β* exacerbated gastritis and accelerated cancer formation [125], whereas *Il1r*^−/−^ mice were protected [126]. Similarly, the lack of IL-1R helped to protect mice from MAC-induced fibrosarcoma, and the same result could be observed in *Nlrp3*^−/−^ or *Caspl*^−/−^ mice; this may be related to NLRP3 inhibiting the function of NK cells [127]. Furthermore, the activation of AIM2-dependent inflammasomes in mice models of spontaneous pancreatic cancer was shown to contribute to pancreatic carcinogenesis [128]. However, in contrast, deleting the *Aim2* [129], *Asc* [129, 130], *Nlrp3* [131, 132], *Nlrc4* [133], *Casp1* [132, 133], or *Il-18r* [134] genes invariably exacerbated the burden of AOM/DSS-induced colorectal cancer in mice. Additionally, in a recent study, *Mefv (*or *pyrin)*^−/−^ mice were similarly unable to limit the extent of inflammation due to the restricted activation of inflammasomes and IL-18 maturation, resulting in increased tumor susceptibility [135]. These results all suggest that IL-18 has a protective role, as the lack of IL-18 leads to the loss of epithelial integrity in mice, exacerbating inflammation and accelerating inflammation-induced colorectal carcinogenesis [131, 134, 135]. Conversely, supplementation with IL-18 or implantation of wild-type myeloid cells reduced tumor burden [135, 136]. IL-18 promoted CD8^+^ T cells to rebuild the intestinal epithelial barrier and produced IFN-*γ* to play a protective role [136]. Moreover, the protective effect of IL-18 could also be partially explained by the role of IL-22; in essence, the colorectal tissue injury sensed by NLRP3 or NLRP6 inflammasomes led to the IL-18-dependent downregulation of the IL-22-binding protein (IL-22BP), which increased the proportion of IL-22 and provided protection during the peak of injury [137]. Notably, IL-22 can also promote tumor development if it is not controlled during the recovery phase of inflammation [137].

## 7. Summary and Prospects

**Figure 2:**
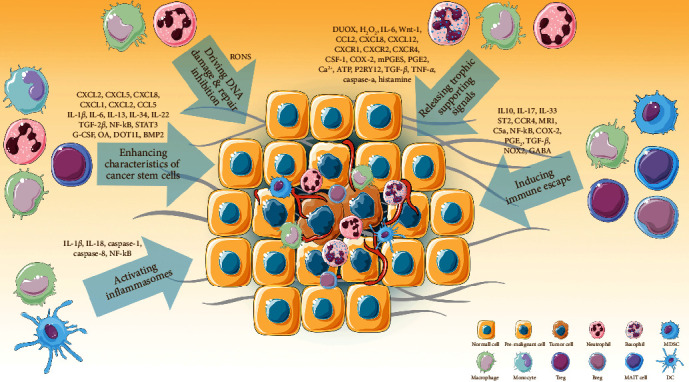
Tumor-promoting immune microenvironment in the early stages of tumorigenesis. Immune cell-mediated tumor-promoting mechanisms involve driving DNA damage and repair inhibition, releasing proliferation signals, inducing immune escape, enhancing characteristics of cancer stem cells, and activating inflammasomes. DUOX, dual oxidase; mPGES, microsomal prostaglandin E synthase; P2RY12, purinergic receptor P2Y12; OA, osteoactivin; ST2, suppression of tumorigenicity 2; MR1, MHC class I-related protein 1; NOX2, NADPH oxidase 2; GABA, gamma-aminobutyric acid; STAT3, signal transducer and activator of transcription 3; G-CSF, granulocyte colony-stimulating factor; DOT1L, disruptor of telomeric silencing 1-like; BMP2, bone morphogenetic protein 2; MDSC, myeloid-derived suppressor cell; Treg, regulatory T cell; Breg, regulatory B cell; MAIT cell, Mucosal-associated invariant T cell; DC, dendritic cell.

We have clearly understood the inevitable link between chronic inflammation and increased cancer risk, as well as the tumor-promoting mechanism of immune cells in early tumorigenesis (Figure 2); therefore, terminating inflammation in the early stages should always be prioritized to prevent tumor formation. However, more often than not, these inflammations persist precisely because we lack the means to address them. Overproduction of RONS is an important factor in inflammation-induced tumor initiation; thus, inhibiting RONS overproduction and the scavengers of RONS or reducing oxidative stress may have considerable potential to prevent tumor initiation. Notably, the results of several clinical studies have demonstrated that antioxidant application and supplementation can reduce the risk of pancreatic cancer [138] and lung cancer [139]. Additionally, long-term use of NSAIDs also reduces cancer incidence and improves cancer prognosis [140], which may arise from the blockade of the trophic effects of COX-2 and PGE_2_. The suppressive TiME in the early stages of tumorigenesis should also be taken into account. Schietinger et al. found that tumor-specific CD8^+^ T dysfunction may be established early during the premalignant phase of tumorigenesis in a reversible state [141]. Therefore, early intervention to avoid its conversion to a fixed state is essential. While tumor immunotherapy has been highly anticipated by researchers worldwide in recent decades, the discovery of tumor checkpoints in particular, including PD-L1, CTLA-4, CD47, and the Siglec family, is a landmark advance in tumor immunotherapy. However, we have not adequately examined immunosuppression in early tumorigenesis, partly because we have difficulty grasping the timing of the formation of COOs or premalignant cells; blindly blocking these immune cells can result in aggravated infections, severe autoimmunity, or even induced tumor formation. As indicated by the findings of Gong et al. [54] and Huber et al. [137], the function of immune cells and cytokines during the inflammatory phase prior to tumorigenesis varies. Similarly, while neutrophils have been largely thought to promote inflammation-induced colorectal tumor progression [142], a new study suggests that neutrophils can limit tumor formation, proliferation, and invasion and that neutrophil-depletion accelerates tumorigenesis [90]. With regard to patients in the chronic inflammatory or early tumor stages, administering drugs widely to a large population is inappropriate, regardless of the variable role played by these immune cells in the TME. Capturing the timing of cellular function transition from cancer suppression to cancer promotion has important implications for further steps in research and therapy. New technologies, such as single-cell sequencing, may facilitate the further analysis of the status and function of immune cells by cell subpopulation. The role of inflammasomes in tumorigenesis is controversial at present. Therefore, the blind use of inflammasome-targeted therapies, especially in patients with colorectal cancer, may not lead to a good prognosis. Meanwhile, the role of CSCs in tumorigenesis and recurrence cannot be ignored. Immunity and inflammation are key factors in the activation of CSCs, and anti-inflammatory therapy seems to reduce the recurrence rate of cancer after surgery [143], but further research is needed. Furthermore, the induction of differentiation of these cells with multidifferentiation potential to normal cells may be an idea.

## Figures and Tables

**Table 1:** The effects of inflammasome activation in mouse tumorigenesis models.

Tumor type	Gene deletion	Mechanism	Effect	Reference
Gastric cancer	*Asc* ^−/−^	Caspase-1↓, NF-*κ*B activity↓, mature IL-18↓, caspase-8-like apoptosis↑	Suppressive	[123]
	*Il18* ^−/−^	Mature IL-18↓, caspase-8-like apoptosis↑	Suppressive	[123]
	*Aim2* ^−/−^	IL-11↓, STAT3 activation↓	Suppressive	[124]
	*Il1r* ^−/−^	Decrease of binding to IL-1*β*	Suppressive	[125, 126]
Fibrosarcoma	*Il1r* ^−/−^, *Nlrp3*^−/−^, *Caspl*^−/−^	Inhibition of NK cells	Suppressive	[127]
Pancreatic cancer	*Aim2* ^−/−^	HMGB1↓	Suppressive	[128]
Colorectal cancer	*Aim2* ^−/−^	DNA-PK-mediated Akt activation↑	Promotive	[129]
	*Asc* ^−/−^	DNA-PK-mediated Akt activation↑, IL-1*β*, and IL-18↓	Promotive	[129, 130]
	*Nlrp3* ^−/−^	IL-1*β* and IL-18↓	Promotive	[131, 132]
	*Nlrc4* ^−/−^		Promotive	[133]
	*Casp1* ^−/−^	IL-1*β* and IL-18↓	Promotive	[132, 133]
	*Il18* ^−/−^, *Il-18r*^−/−^	IL-18↓, decrease of binding to IL-18	Promotive	[134]
	*Mefv*/*pyrin*^−/−^	IL-18↓, IL-6↑	Promotive	[135]
